# Paediatric Cushing’s disease: Epidemiology, pathogenesis, clinical management and outcome

**DOI:** 10.1007/s11154-021-09626-4

**Published:** 2021-01-30

**Authors:** Rosario Ferrigno, Valeria Hasenmajer, Silvana Caiulo, Marianna Minnetti, Paola Mazzotta, Helen L Storr, Andrea M Isidori, Ashley B Grossman, Maria Cristina De Martino, Martin O Savage

**Affiliations:** 1 Dipartimento di Medicina Clinica e Chirurgia, Federico II University, Naples, Italy; 2 Department of Experimental Medicine, Sapienza University of Rome, Rome, Italy; 3 Primary care Paediatrician, Local Health Unit of Brindisi, Brindisi, Italy; 4 Centre for Endocrinology, William Harvey Research Institute, Barts and the London School of Medicine & Dentistry, London, UK; 5 Royal Free Hospital ENETs Centre of Excellence, London, UK; 6 Oxford Centre for Diabetes, Endocrinology and Metabolism, University of Oxford, Oxford, UK; 7 Centre for Endocrinology, William Harvey Research Institute, Charterhouse Square, London, EC1M 6BQ UK

**Keywords:** Hypercortisolism, Paediatric, Cushing’s, Cortisol, Pituitary

## Abstract

Cushing’s disease (CD) is rare in paediatric practice but requires prompt investigation, diagnosis and therapy to prevent long-term complications. Key presenting features are a change in facial appearance, weight gain, growth failure, virilization, disturbed puberty and psychological disturbance. Close consultation with an adult endocrinology department is recommended regarding diagnosis and therapy. The incidence of CD, a form of ACTH-dependent Cushing’s syndrome (CS), is equal to approximately 5% of that seen in adults. The majority of ACTH-secreting adenomas are monoclonal and sporadic, although recent studies of pituitary tumours have shown links to several deubiquitination gene defects. Diagnosis requires confirmation of hypercortisolism followed by demonstration of ACTH-dependence. Identification of the corticotroph adenoma by pituitary MRI and/or bilateral inferior petrosal sampling for ACTH may contribute to localisation before pituitary surgery. Transsphenoidal surgery (TSS) with selective microadenomectomy is first-line therapy, followed by external pituitary irradiation if surgery is not curative. Medical therapy to suppress adrenal steroid synthesis is effective in the short-term and bilateral adrenalectomy should be considered in cases unfit for TSS or radiotherapy or when urgent remission is needed after unsuccessful surgery. TSS induces remission of hypercortisolism and improvement of symptoms in 70–100% of cases, particularly when performed by a surgeon with experience in children. Post-TSS complications include pituitary hormone deficiencies, sub-optimal catch-up growth, and persisting excess of BMI. Recurrence of hypercortisolism following remission is recognised but infrequent, being less common than in adult CD patients. With experienced specialist medical and surgical care, the overall prognosis is good. Early referral to an experienced endocrine centre is advised.

## Introduction

Cushing’s disease (CD), defined as hypercortisolism caused by excess adrenocorticotropic Hormone (ACTH) secretion by a pituitary corticotroph adenoma [1], presents rarely in the paediatric age range. However, this serious disorder requires early diagnosis and specialised management because the morbidity related to chronic hypercortisolism in paediatric patients is considerable. Its rarity, having an incidence approximately 5% of that seen in adults [2], has resulted in paediatric endocrinologists having limited experience in diagnosis or therapy in this essentially adult disorder. Although the principles of investigation and therapy should be conducted in paediatric facilities and are based on adult experience, management of paediatric CD by paediatricians in isolation is not recommended. The active cooperation and discussion with adult endocrinologists, including consultation on invasive radiological and neurosurgical techniques, is beneficial to patient care.

In terms of aetiology, new genetic findings related to mutations of deubiquitinase genes involved in pituitary adenoma growth and secretion of ACTH have questioned the traditional view that corticotroph adenomas are essentially sporadic and monoclonal in origin [3]. The presenting features of paediatric CD resemble those in adults; however, some notable differences have been recognised such as male predominance in prepubertal patients, disturbance of growth and puberty, predominance of microadenomas, poor prediction by pituitary MRI of tumour visibility and, in terms of therapy, more rapid control of hypercortisolism following pituitary radiotherapy (RT) [4].

These and other features of the epidemiology, pathogenesis, diagnosis, therapeutic approach and long-term outcome will be discussed in this review. The authors comprise both paediatric and adult endocrinologists, consistent with our message of close collaboration between the two disciplines for the optimal care of patients with paediatric CD.

## Epidemiology

Endogenous Cushing’s syndrome (CS) is rare in children and adolescents and is relatively uncommon in adults, with an incidence of 0.7–2.4 per million in the general population per year [1, 5]. Two studies from Denmark and Spain reported incidences of endogenous CS as 2 and 2.4 cases respectively per million inhabitants per year [6, 7]. The commonest cause of endogenous CS in all age groups is Cushing’s disease (CD). At all ages, corticotroph microadenomas are the commonest cause of CD [1]. Corticotroph macroadenomas occur in only ~10% of adult CD cases and are even rarer in children [4, 8].

The annual incidence of CD in Sweden during the years 1987 to 2013 was reported as 1.6 (1.4–1.8) cases per million. Mean (± SD) age at diagnosis was 43 ± 16 years. Of 534 patients, 32 (6%) were younger than 18 years at diagnosis: 17 (53%) girls and 15 (47%) boys [2]. CD accounts for ~70% of adult-onset [1, 9] and 75–80% of paediatric-onset endogenous CS [10, 11]. An estimated 1 to 1.5 per million children are affected each year by CS; of those 75–80% are caused by an ACTH-secreting pituitary tumour (CD) [12]. Corticotroph adenomas are reported to constitute 54.8% of all pituitary adenomas in the ages 0–11 years and 29.44% in the ages 12–17 years [13]. In children aged less than 5 years, CD is extremely rare — the most common causes of CS in this age group are adrenocortical adenoma or carcinoma and bilateral adrenal hyperplasia [12]. The mean age of CD presentation, in several large paediatric series comprising 41–182 children, was 12.3–14.1 years [14].

## Pathogenesis

The majority of ACTH-secreting adenomas are monoclonal and sporadic [15]. Rarely, CD may occur in the context of a genetic neoplasia syndrome; however the majority of paediatric corticotroph adenomas are not caused by germline genetic defects [16].

### *MEN1* mutations

Multiple endocrine neoplasia (MEN) 1 is an autosomal dominant disorder characterised by neuroendocrine neoplasia arising mainly in parathyroid glands, pancreatic islet cells, and the anterior pituitary gland. CD can very rarely be part of MEN 1, due to mutations in the tumour suppressor gene *MEN1*, which encodes a 610-amino acid protein, menin. Pituitary adenomas occur in 20–50% of MEN 1 cases, mostly somatotroph or lactotroph in origin, but corticotroph adenomas have rarely been described [17, 18]. In a cohort of 74 paediatric cases with sporadic CD, genetic screening identified an *MEN1* mutation in 1 patient [19]. Additionally, 4 paediatric patients aged 11–14 years had CD associated with a family history of genetically-confirmed MEN1 (*n* = 2), clinical features of MEN1 (*n* = 1) or a TSC2 mutation-positive tuberous sclerosis (n = 1) [19]. Genotyping demonstrated that the two patients were carriers of their familial MEN1 mutations and of the 4 patients with suspected genetic syndromes and CD, only 2 syndromic patients who had relatives with genetically-confirmed MEN 1 syndrome had *MEN1* mutations [19].

### FIPA

Familial isolated pituitary adenomas (FIPA) is a genetic condition characterised by pituitary adenomas in the absence of other clinical features. In 20% of FIPA families, mutations in the aryl hydrocarbon receptor-interacting protein (*AIP*) gene have been identified. However, CD very rarely occurs in FIPA and germline AIP mutations are infrequently found in patients with CD [20]. In 73 paediatric CD cases, only 1 (1.4%) was found to have an AIP mutation [19].

### Deubiquitinase gene mutations

#### USP8 mutations

Studies using next generation sequencing on the molecular origin of pituitary adenomas have started to unveil the genetic basis of CD. The most frequent somatic mutations in paediatric CD have been detected in the *USP8* gene that was identified in 31–63% of corticotroph adenomas [3, 21, 22]. The *USP8* gene encodes a deubiquitinase, which regulates the stability of tyrosine kinase receptors, such as the epidermal growth factor receptor (EGFR) [3]. The deubiquitination of EGFR prevents its lysosomal degradation, allowing downstream signalling pathways to function in an active state [23]. In a study from the NIH [22], 5 different *USP8* mutations (3 missense, 1 frameshift, and 1 in-frame deletion) were identified in 13/42 CD patients (31%), all of them located in exon 14 at the described mutational hotspot, affecting the 14–3-3 binding motif of the protein. Patients with somatic mutations were older [mean 15.1 ± 2.1 vs 13.1 ± 3.6 years, *P* = 0.03]. Biochemical variables of hypercortisolism and ACTH, as well as tumour size and frequency of invasion of the cavernous sinus, were not different between *USP8-*mutated and non-mutated subjects. However, patients harbouring somatic *USP8* mutations had a higher likelihood of recurrence of CD following transsphenoidal surgery (TSS) compared with patients without mutations (46.2% vs 10.3%, *P* = 0.009).

However, the major driver mutations in *USP8* wild-type tumours remain elusive. *POMC* gene transcription is stimulated by high EGFR levels, with a subsequent increase in plasma ACTH levels [3]. *USP8*-mutated corticotroph adenoma cells showed higher EGFR transcript and immunoreactivity in human studies [23]. A 16 year old female patient with a *de novo* germline *USP8* mutation with recurrent CD and multiple other medical problems was recently reported [24]. Associated features were developmental delay, dysmorphic features, ichthyosiform hyperkeratosis, chronic lung disease, chronic kidney disease, hyperglycemia, dilated cardiomyopathy with congestive heart failure, and previous history of hyperinsulinism and partial growth hormone (GH) deficiency (GHD). She underwent transsphenoidal surgery and improved, however her CD recurred [24]. Heterozygous somatic single point mutations have been reported exclusively in CD. The prevalence of *USP8* mutations is higher in female than in male patients with CD, possibly related to oestrogenic action on *USP8*-mutated cells [21]. Next generation sequencing is facilitating the clarification of the genetic basis of CD. The presence of somatic *USP8* driver mutations in a significant portion of corticotroph adenomas represents a novel and unique mechanism leading to ACTH excess. It is possible that inhibition of USP8 or its downstream signalling pathways could represent a new therapeutic approach for the management of CD [25].

#### *USP48* and *BRAF* mutations

Recurrent mutations were reported in an additional deubiquitinase gene, *USP48* (predominantly encoding p.M415I or p.M415V) in 21/91 paediatric subjects with corticotroph adenomas and in the *BRAF* gene (encoding p.V600E) in 15/91 subjects with wild-type *USP8* [26]. Similar to *USP8* mutants, both *USP48* and *BRAF* mutants enhance the promoter activity and transcription of the *POMC* gene providing a potential mechanism for ACTH overproduction [26]. However, these interesting findings require confirmation.

### CABLES1 mutations

Another gene related to the pathogenesis of ACTH-secreting adenomas is the *CABLES1* (Cdk5 and ABL enzyme substrate 1) gene (18q11.2), which negatively regulates cell cycle progression in response to glucocorticoids. Indeed, the CABLES1 protein expression is lost in around half of corticotroph adenomas [27]. In a cohort of 146 paediatric CD cases, putative *CABLES1* mutations were identified in 2 sporadic female patients [28].

### Additional mutations in CD patients

In adults, cyclin E is over-expressed in corticotroph tumours, and mutations in *cyclin E* (*CCNE*), *EGFR*, *CMPtk*, and *LAPTM4B* gene have been related to ACTH-secreting tumours [29]; however, these defects are rare in the paediatric CD population [30]. Germline loss-of-function *CDKN1B* gene variants, which are known to cause MEN type 4 in adults, have now been shown to rarely cause sporadic CD without the MEN syndrome in children with CD [31].

## Clinical presentation

Early recognition of the presenting clinical signs and symptoms of CD is essential for prompt diagnosis and treatment. The definition of ‘paediatric’ CD varies in different countries, being <18 years in the UK [4], <18 years in the USA [32] and < 20 years in India [33]. Mean ages at diagnosis were 13.1 [34], 12.9 [35], 12.3 [4] and 14.9 years [33]. CD is rare below the age of 5 years. Key clinical findings in paediatric CD patients are shown in Table 1**.**

**Table 1:** Frequency of clinical findings at diagnosis of paediatric Cushing’s disease

	Devoe 1997 [34]	Shah 2011 [33]	Storr 2011 [4]	Lonser 2013 [36]	Guemes 2016 [37]
Total number of patients	42	48	41	200	16
Mean age / median age (range)	13.4 y^a^(6.5–18)	14.85^b^ ± 2.5 y (9–19)	12.3 ^b^ ± 3.5 y (5.7–17.8)	10.6 ^b^ ± 3.6 y (4–19)	10 y^a^(7–15.5)
Clinical symptoms and signs (%)					
Weight gain	92	98	98	93	94
Growth retardation	84	83	100	63^c^	63
Short stature		56			
Facial changes	46	98	100	63	
Irregular menses (females)				49^d^	
Osteopaenia	74				
Fatigue or weakness	67		61	48	38
Hirsutism	46		59	56	38
Virilization			76		
Psychiatric disorders	44^e^			31^f^	
Mood changes		46	59^g^		44^h^
Headache	26		51	38	
Striae	36	58	49	55	44
Hypertension	63	71	49	36	50
Acne	46		44	47	50
Pubertal delay or arrest	60				19
Early secondary sexual development					31
Easy bruising	28	17		25	19
Dorsal cervical or supraclavicular fat pad	28			69	
Hyperpigmentation					13
Acanthosis nigricans		75		32	
Muscle weakness		48			
Sleep disturbances					19
Glucose intolerance or diabetes		25		7	
Bone fractures				4	
Hypokalemia					6
Infection		15			

Y, years; ^a^ median age; ^b^ mean age; ^c^ pre-pubertal patients (*n* = 91) showed growth retardation in 85% of cases, post-pubertal patients (*n* = 109) showed growth retardation in 44% of cases; ^d^ primary or secondary amenorrhea; ^e^ compulsive behaviour; ^f^ depression, anxiety, mood swings; ^g^ emotional lability/depression; ^h^ mental changes changes/poor school performance

### Gender and its possible effect on severity

Gender distribution at the diagnosis of CD was analysed in 50 patients aged from 6 to 30 years [38]. In 25 of the patients aged 6–18 years, there were 17 males (68%) and 8 females (32%) contrasting with 4 males (16%) and 21 females (84%) in the 25 subjects aged 18 to 30 years. CD diagnosed before age 18 years occurred predominantly in males, contrasting with female dominance at 18 years or older (*P* = 0.0003). There was also a significant difference χ^2^ in sex distribution depending on pubertal status (*P* = 0.0002). In 17 prepubertal CD patients from the same institution, 76% were male and 24% female [39]. Male predominance (89%) was also seen in prepubertal patients from the NIH [40]. In CD patients presenting during puberty an equal sex incidence (50% males) was reported [38]. However, a larger series of 102 patients from the NIH showed an equal gender distribution in both prepubertal and pubertal subjects [35]. In a series of 183 adult CD patients, aged from 18 to 95 years, there were 144 (77%) females and 39 (23%) males [4]. The origin of the male predominance before puberty is not currently understood.

Clinical and biochemical abnormalities have been reported to be more severe in adult males with CD, who also presented at a younger age than adult females [41] Storr et al. found found no gender differences in clinical or biochemical features in 50 CD subjects aged 6 to 30 years [38]; however in 102 paediatric patients from NIH, male subjects presented with higher BMI, possibly shorter height and increased plasma ACTH, suggesting a more aggressive form of CD in boys [35].

### Facial changes, growth and puberty

Facial changes, showing the typical Cushingoid appearance, are very common in children with CD. Swelling of the face and change in appearance, clearly seen in family photos, were present in 100% of patients reported by Storr et al. [4], and has been documented in all large paediatric CD series [33, 36]. These changes are very distressing for the patients, who may try to lose weight to decrease them. However, they may not be recognised by general practitioners or paediatricians as being pathological, which can result in long intervals between onset of symptoms and diagnosis, reported as 3 years (range 0.25–7) in 50 patients [40] and 2.5 years (range 0.3–6.6) in 43 patients [42].

Chronic hypercortisolism causes growth failure, usually associated with abnormal weight gain. Short stature is not always present, being reported in 42% and 56% of cases [32, 33]; however, when height velocity has been calculated, it is subnormal [42]. Bone age is typically delayed at diagnosis [43].The most striking auxological finding is the contrast between height SDS and BMI SDS [33, 40], which was notably absent in subjects with simple obesity, where height SDS is typically above average [44]. A further clinical abnormality related to hypercortisolism and increased adrenal androgens is disturbance of the normal harmony of secondary sexual development. Increased virilisation, shown by advanced Tanner stage of pubic hair growth, is frequently present in prepubertal patients and was positively associated with increased serum androstenedione, DHEA-S and testosterone SDS values and decreased SHBG SDS compared to non-virilised subjects [45]. Hirsutism, acne and purple striae are also common, particularly in older patients. Gonadotrophin secretion is suppressed by hypercortisolism contributing to the imbalance of puberty development with low Tanner stage of breast development and impaired testicular growth combined with advanced stages of pubic hair [45].

### Psychiatric disturbances, additional features and contrasts between paediatric and adult CD

Mood changes, depression and emotional lability are common in children with CD [36, 42]. More rarely, acute psychosis has been documented which may be an indication for emergency bilateral adrenalectomy [46, 47]. Muscle weakness, osteopaenia, polycythaemia and headache are also common. A comparison between features in paediatric compared with adult CD patients [4] showed that weight gain was present in 99% of the paediatric subjects (mean BMI SDS at diagnosis 2.7; range 0.0–9.2) in contrast to 65% of the adult subjects (*P* < 0.001). All paediatric patients reported facial changes compared with 81% of the adults (*P* < 0.01). Fatigue and emotional lability or depression were commoner in paediatric compared with adult subjects (*P* < 0.0001 and *P* < 0.006 respectively). Hypertension was the most common additional feature in adult patients (77%) but was less common in paediatric patients (49%, *P* < 0.0009).

## Diagnostic investigations

Firstly, in paediatric patients with suspected CD, ingestion or administration of exogenous corticosteroids should be promptly excluded by a careful history before biochemical evaluation. At the initial assessment, clinical data on auxological parameters, puberty stage, excessive virilisation and bone age determination should be obtained.

### Confirmation of hypercortisolism

Documentation of hypercortisolism is the first step in the diagnostic process. Essentially, three tests that can be used for this purpose: 24-h urinary free cortisol (UFC), late-night sleeping salivary/serum cortisol and dexamethasone-suppression testing. Because none of these tests has 100% diagnostic accuracy (Table 2) and each test has some limitations, multiple tests are usually needed to confirm hypercortisolism. Recently, a study including paediatric patients (58% of the cohort less than 18 years) showed that proximal hair cortisol could also be a good marker reflecting hypercortisolism [57], but further studies are needed to confirm the diagnostic value of this technique.

**Table 2:** Diagnostic tests for paediatric hypercortisolism

Test	AUTHOR	SENSITIVITY	SPECIFICITY	Patients age	TEST CHARACTERISTICS	**CUT-OFF**
Urinary free cortisol excretion		86–100%	90–100%			
	Batista 2007 [48]	88% (92/105)	90%(18/20)^e^	12 y^a^ (5–17)	24-h urine collection(s)	>70 μg/m^2^/day
	Bickler 1994 [49]	100% (6/6)		15.7 y^b^ (11.8 mo-17)		
	Devoe 1997 [34]	86% (25/29)		13.4 y^a^(6.5–18)		>80 μg/m^2^/day
	Gafni 2000 [50]	93% (13/14)	100% (53/53)^f^	5–16 y		>72 μg/m^2^/day
	Guemes 2016 [37]	94%(17/18)		10 y ^a^(7–15.5)		>100 μg/day ^c^
	Leinung 1995 [51]	100%(21/21)		15.5 y^b^(10.1–18.9)		>108 μg/day ^c^
	Shapiro 2016 [52]	87% (34/39)	100%(19/19)^e^	11.7 y^b^ (5.7–16.9)		Various^d^
	Wędrychowicz 2019 [53]	100% (4/4)		11.7y ^b^(7–15)		>55 μg/m^2^/day
		**11–100%**	/			
Low-dose dexamethasone suppression test	Bickler 1994 [49]	11% (1/9)		15.7 y^b^ (11.8 mo-17)	25 μg/kg at midnight (maximum dose 1 mg)	<50% serum cortisol baseline
	Guemes 2016 [37]	100%(20/20)		10 y^a^ (7–15.5)	20 μg/kg/day every 6 h for 48 h	>1.8 μg/dl^c^
	Hsu 2010 [54]	67%(2/3)		11.7 y^b^ (10.9–12.9)		
	Storr 2011 [4]	92%(35/38)		12.3y^b^(5.7–17.8)	0.5 mg every 6 h for 48 h (30 μg/kg/day in children <30 kg)	
	Wędrychowicz 2019 [53]	75%(3/4)		11.7 y ^b^(7–15)	1 mg at 11.00 p.m.	
		93–100%	95–100%			
Late night Cortisol	Batista 2007 [48]	99%(104/105)	100%(20/20)^e^	12 y^a^(5–17)	Midnight serum cortisol	>4.4 μg/dl
	Wędrychowicz 2019 [53]	100%(4/4)		11.7y ^b^(7–15)		
	Davies 2005 [55]	100%(17/17)		12.2 y^b^ (6.4–16.6)		>1.8 μg/dl ^c^
	Guemes 2016 [37]	100% (38/38)		10 y^a^(7–15.5)		≥3.2 μg/dl
	Shah 2011 [33]	94%(45/48)		14.8 y^b^(9–19)		≥5 μg/dl
	Gafni 2000 [50]	93%(13/14)	100%(60/60)^f^	5–16 y	Midnight salivary cortisol	>7.5 nmol/l
	Martinelli 1999 [56]	100%(11/11)	95% (20/21)^g^	10.2 y^b^(1–16)		>7.7 nmol/l

Studies describing at least 10 paediatric CD patients were selected. ^a^ median age; ^b^ mean age; ^c^ UFC converted from nmol/m^2^/day to μg/m2/day or from nmol/day to μg/day, dividing by 2.76; Serum cortisol converted from nmol/l to μg/dl dividing by 27.6; ^d^ Until 1995 > 240 nmol/day; between 1995 and February 2012 > 340 nmol/day, since February 2012 > 124 nmol/day (liquid chromatography-mass spectrometry method); ^e^ Children referred for evaluation of CS; ^f^ Healthy children; ^g^ Obese children. Mo = months; Y = years

### Urinary free cortisol excretion

UFC has been described as a screening test for hypercortisolism in paediatric patients in various studies [34, 36, 37, 48–53]. UFC should always be corrected for body surface area (μg/m^2^ per 24 h). Averaging 24 h UFC values over two or three days provided better accuracy than a single measurement [48, 52]. Nevertheless, the sensitivity of this test analysed in several large series of paediatric patients with CS due to various causes was less than 90% [34, 48, 52], and therefore 24-h UFC alone is not an ideal screening tool. The main advantages are non-invasiveness and the possibility to collect the 24-h samples at home. However, collection may be difficult in the youngest children. Moreover, a physiological increase in the excretion of UFC can occur in girls during the peri-menarche phase [58]. Evaluation of 17-hydroxysteroid excretion offers no benefit in the workup for paediatric CS [48].

### Dexamethasone suppression test (DST)

Diagnosis of hypercortisolism can be made on the basis of failure to suppress serum cortisol to <1.8 μg/dl (<50 nmol/l) during a DST. This test has not been evaluated extensively in children and is carried-out essentially by two techniques:

- Overnight DST: 25 μg/kg at 11 p.m./midnight (maximum dose 1 mg) with sampling for serum cortisol at 09.00 h [49, 53];
- Low-dose DST (LDDST): 20–30 μg/kg/day in children <30 kg (maximum dose 2 mg/day) divided in 0.5 mg doses every 6 h, given at 09.00, 15.00, 21.00 and 03.00 h for 48 h [4, 33, 37, 54].

The LDDST has higher sensitivity than the overnight test (Table 2). Increasing the serum cortisol cut-off from 1.8 to 5 μg/dL lowered the sensitivity value [33]. It has been also reported that in the LDDST, cortisol suppression correlated with that during a HDDST (r = +0.45, *p* < 0.05) with >30% cortisol suppression in the LDDST predicting that change in the HDDST and hence CD [59].

### Late night cortisol

Loss of cortisol circadian rhythm is a hallmark of CD. In normal subjects serum cortisol is <50 nmol/l during sleep at midnight, as is salivary cortisol. This can be evaluated by serum and salivary late-night sleeping cortisol. Assessment of elevated midnight serum cortisol gives high sensitivity (94–100%) and specificity (100%) for hypercortisolism in paediatric CD patients; however, different cut-offs (from 1.8 to 5 μg/dl) have been proposed (Table 2) [33, 37, 48, 53, 55]. The child should be cannulated before sleeping and not approached for a venipuncture while asleep. Measurement of salivary cortisol late at night represents a non-invasive, easy and cost-effective method. To date, the use of late-night salivary cortisol in children has been evaluated only in two studies comparing paediatric CD patients with healthy children [50] and obese controls [56], showing high sensitivity (93–100%) and specificity (95–100%) of this test.

### Confirmation of pituitary aetiology

After the diagnosis of hypercortisolism, it is essential to distinguish ACTH-dependent from ACTH-independent CS. Diagnostic investigations are described in Table 3**.**

**Table 3:** Tests for confirmation of Cushing’s disease

TEST	AUTHOR	SENSITIVITY	SPECIFICITY	AGE	TEST CHARACTERISTICS	DIAGNOSTIC CUT-OFF
ACTH		70–100%	100%			
	Batista 2007 [48]	70%(56/80)	100%(20/20)^c^	12 y^a^ (5–17)	Morning ACTH	>29 pg/ml
	Bickler 1994 [49]	44% (4/9)		15.7 y^b^ (11.8 mo-17)		> 100 pg/ml
	Cirak 1999 [60]	100% (3/3)		13.7 y^a^(13–15)		>60 pg/ml
	Dias 2010 [39]	100% (17/17)		9.4 y^a^(5.7–14)		>10 pg/ml
	Guemes 2016 [37]	92% (11/12)		10 y^a^ (7–15.5)		>15 pg/ml
CRH TEST		74–100%	/			
	Batista 2007 [48]	74% (59/80)		12 y^a^(5–17)	1 μg/kg	Cortisol increase≥20% at 30 or 45 min
		81% (64/79)				ACTH increase≥35% at 15 or 30 min
	Storr 2011 [4]	92% (36/39)		12.3 y^b^(5.7–17.8)		Cortisol increase ≥20%
	Wędrychowicz 2019 [53]	100% (3/3)		11.7y ^b^(7–15)		Cortisol increase ≥20% + ACTH increase ≥35%
High-dose dexamethasone suppression Test (HDDST)		67–97.5%	43–100%			
	Batista 2007 [48]	97.5% (77/79)	100% (23/23)^c^	12 y^a^(5–17)	120 μg/kg (max 8 mg) at 11:00 p.m.	Cortisol decrease≥ 20% from baseline
	Bickler 1994 [49]	67% (6/9)		15.7 y^b^ (11.8 mo-17)	8 mg/1.73 m^2^ (max 8 mg) at 11 p.m.	Cortisol decrease>10% from baseline
	Cirak 1999 [60]	100%(3/3)		13.7 y^a^(13–15)	8 mg at 11 p.m.	
	Storr 2011 [4]	93%(26/28)		12.3 y^b^ (5.7–17.8)	120 mg/kg/day for 48 h	
	Guemes 2016 [37]	90%(9/10)		10 y^a^(7–15.5)	80 μg/kg/day every 6 h for 48 h (maximum 2 mg)	
	More 2011 [61]	83%(10/12)	43% (3/7)^d^	16.5 yb (11–20)	120 μg/kg/day (maximum dose: 8 mg)	
	Shah 2011 [33]	74%(35/48)		14.8 y^b^(9–19)		
	Lonser 2013 [36]	90% (151/167)		10.6 y^a^ (4–19)	NA	Cortisol decrease>75% from baseline
Magnetic Resonance Imaging of the sellar region		16–100%	50–67%			
	Batista 2005 [62]	24% (6/25)	67% (2/3)^e^	12 y^a^(6–17)	Precontrast SPGR MRI	Adenoma detection
		75% (18/24)	50%(2/4)^e^		Postcontrast SPGR MRI	
		16% (4/25)	67% (2/3)^e^		Precontrast SE MRI	
		21% (5/24)	50%(2/4)^e^		Postcontrast SE MRI	
	Cirak 1999 [60]	67% (2/3)		13.7 y^a^(13–15)	NA	
	Das 2007 [63]	80%(8/10)		15 y^a^(12–17)	Dynamic MRI	
	Gazioglu 2019 [64]	70% (7/10)		14.8 y^b^(5–18)	NA	
	Hsu 2010 [54]	100%(3/3)		11.7 y^b^(10.9–12.9)		
	Kanter 2005 [65]	67% (22/33)		13 y^b^(5–19)		
	Shah 2011 [33]	71% (34/48)		14.8 y^b^ (9–19)		
	Storr 2011 [4]	55% (21/38)		12.3 y^b^ (5.7–17.8)		
	Wędrychowicz 2019 [53]	100% (4/4)		11.7y b (7–15)		
Bilateral inferior petrosal sinus sampling (BIPSS)		60–99%	NA			
	Batista 2006 [8]	90% (83/92)		13 y^b^ (5.3–18.7)	Diagnosis of CD	Basal C/P ACTH ratio > 2
		97% (88/92)				CRH stimulated C/P ACTH ratio > 3
	Storr 2011 [4]	76% (22/29)		12.3 y^b^(5.7–17.8)		Basal C/P ACTH ratio > 2
		86% (25/29)				CRH stimulated IPS/P ratio > 3
	Lonser 2013 [36]	99% (139/140)		10.6 y^a^ (4–19)		NA
	Shah 2011 [33]	61.5% (8/13)		14.8 y^b^ (9–19)		Basal C/P ACTH ratio > 2
	Batista 2006 [8]	60% (35/58)		13 y^b^ (5.3–18.7)	Adenoma lateralization	Inter-petrosal ratio > 1.4
	Storr 2011 [4]	76% (25/33)		12.3y^b^(5.7–17.8)		

^a^median age; ^b^ mean age; ^c^ Children with adrenocortical tumours;^d^ Children with ectopic CS;^e^ comparing the imaging data with the surgical findings; *C/P* Central to peripheral, *CD* Cushing’s disease, *min* Minutes, *mo* Months, *MRI* Magnetic Resonance Imaging, *NA* Not available, *SPGR* spoiled gradient recalled, *y* Years

### Basal ACTH

In paediatric CD, morning plasma ACTH is typically detectable (>5 pg/ml), while patients with primary adrenal diseases showed suppressed ACTH [39, 48]. The largest series using morning ACTH for differentiate patients with ACTH-dependent or independent CS showed that a cut-off of 29 pg/ml had a sensitivity of 70% and a specificity of 100% [48]. Mean plasma ACTH was higher in paediatric patients with ectopic ACTH syndrome (EAS) than with CD, all patients with EAS and 68% of patients with CD displaying ACTH concentrations above the normal range [61]. Although ACTH assay reliability has improved significantly in recent years, basal ACTH levels may show variability due to the circadian modifications and instability after the sample collection.

### CRH stimulation test

To confirm the diagnosis of pituitary-dependent hypercortisolism, a test using intravenous injection of 1 μg/kg CRH (maximum dose 100 μg) is recommended [4, 37, 39, 48, 53]. In CD, the ACTH secreting pituitary adenoma is reported to give an exaggerated response to CRH resulting in an elevated cortisol response [66, 67]. The most used cut-offs for the differential diagnosis of paediatric CD as opposed to ectopic ACTH are a mean percentage increase of 20% above baseline for cortisol values (at 30′ and 45′) and an increase in the mean ACTH concentrations of at least 35% over basal values (at 15′ and 30′) after CRH injection [4, 48, 53]. It is interesting that the paediatric population exhibited a more exuberant cortisol response than adults, enhancing the usefulness of the CRH test to differentiate CD from EAS in children [4, 68]. Finally, desmopressin testing, which also induces an excess ACTH and cortisol response in CD patients, has been used in patients with extremely difficult venous access [68].

### High-dose dexamethasone suppression test (HDDST)

In paediatric patients 80–120 μg/kg (maximum dose 8 mg) of dexamethasone administered in one dose at 11:00 PM or in 4 divided doses, each of 2.0 mg, for 48 h have been described [4, 33, 36, 37, 39, 48, 49, 60, 61, 63]. Although a decrease ≥50% in morning cortisol from baseline has been used as cut-off in most of studies describing CD patients [4, 33, 37, 39, 63], a decrease ≥20% showed the highest sensitivity (97.5%) and also 100% specificity to distinguish CD from adrenal tumours [48]. Paediatric patients with EAS, although extremely rare, may show high degrees of cortisol suppression after HDDST, and therefore this test does not accurately exclude EAS [37, 61]. It should be also noted that in children, HDDST can induce adverse effects, such as transient hypertension or hyperglycaemia [59]. For these reasons and following the diagnostic value of the LDDST, the HDDST is no longer routinely used in some centres [42].

### Pituitary magnetic resonance imaging (MRI)

MRI has replaced other radiological techniques for pituitary visualisation and should be obtained after biochemical confirmation of ACTH-dependent CS. Paediatric CD is predominantly associated with corticotroph microadenomas, usually <6 mm in diameter, which are typically hypodense on MRI and frequently fail to enhance with gadolinium contrast [36, 39]. Therefore, it is mandatory that pituitary MRI should be performed with thin sections and high resolution at specialist tertiary referral centres for CD. Even so, in the largest and latest series of paediatric CD patients, with more than 20 children described in each, MRI correctly identified a pituitary adenoma in only 16–71% of patients [4, 33, 36, 62, 65]. Dynamic magnetic resonance can be helpful in diagnosing microadenomas whereas the routine MRI sequences are equivocal [63]. Also, post-contrast spoiled gradient-recalled (SPGR) acquisition seems superior to conventional MRI and dynamic contrast spin echo MRI in paediatric CD patients to detect smaller adenomas [62].

A formal comparison of MRI results in paediatric (*n* = 39) compared with adult (*n* = 66) CD patients was performed by Storr et al. [4]. Pituitary MRI demonstrated less macroadenomas in paediatric 2% (1/41) compared with 15% (28/183) of adult patients (*P* = 0.04). The appearances were consistent with a microadenoma in 55% (21/38) of paediatric patients compared with 76% (50/66) of adult patients (*P* = 0.045). The percentage concordance of the microadenoma position by imaging compared with the findings at TSS was also lower in children (34%, 13/38) compared with adults (57%, 27/47) (*P* = 0.058).

Consequently, a positive MRI is beneficial and supports the diagnosis of CD and adenoma identification, but the relatively low prediction rate requires the option of more precise adenoma localisation using bilateral inferior petrosal sinus sampling (BIPSS).

### Bilateral inferior petrosal sinus sampling (BIPSS)

Preoperative BIPPS with CRH stimulation (1 μg/kg, max 100 μg) has been suggested in paediatric patients when there is a negative pituitary MRI with confirmed ACTH-dependent hypercortisolism [8]. However, the technique of BIPSS will not have been established in paediatric institutions and should be performed only by experienced radiologists in dedicated centres. Sedation during BIPSS is routinely not required, although general anaesthesia may be necessary in the youngest patients [39]. BIPSS is generally not necessary for confirmation of a pituitary tumour in patients with a positive MRI and biochemical features of CD, because the incidence of incidentalomas in children is minimal [69, 70]. Ectopic ACTH secretion in children is also extremely rare, so the primary aim of BIPSS is to provide the localisation of the microadenoma by demonstrating lateralisation of ACTH secretion. In the largest series of paediatric CD patients, simultaneous BIPSS, with a basal central-to-peripheral ratio > 2 and > 3 after CRH injection, had a sensitivity of 76–99% for the diagnosis of CD [4, 8, 36, 39]. Using the ACTH inter-petrosal ratio > 1.4, the sensitivity to detect lateralisation of an adenoma in the largest series of patients with paediatric CD (>20 cases) was 60–88% [4, 8, 36].

### Cavernous sinus sampling (CSS)

CSS has been proposed as an alternative to BIPSS to improve the diagnostic accuracy in the differential diagnosis and localisation of pituitary adenomas. CSS is an invasive technique, which requires general anaesthesia and heparinisation. To date, only one small study reported CSS in paediatric patients, showing a sensitivity of 100% [64].

## Treatment of paediatric Cushing’s disease

Up to now, no consensus guidelines, specifically for the treatment of paediatric CD, have been published. However, in the 2015 *Endocrine Society Clinical Practice Guidelines* on the treatment of CS, data regarding paediatric CD were included. These guidelines can therefore be considered as the current standard of care for therapy [71]. In paediatric patients, normalisation of cortisol levels, reversal of hypercortisolism-related signs and symptoms, and pituitary adenoma removal are the main treatment goals. In terms of removal of the primary source of excess ACTH secretion, selective adenomectomy through TSS is agreed to be the first-line treatment [72]. Pituitary RT aims to suppress excess ACTH secretion and to inactivate its source. Success of both these treatments will also reverse hypercortisolism and its symptoms [71, 72].

Two additional forms of therapy, namely medical therapy and bilateral adrenalectomy, will not target the primary cause, but can reverse hypercortisolism and the clinical features related to it. In cases of recurrence of hypercortisolism following TSS or external pituitary irradiation, repeat TSS, medical therapy or bilateral adrenalectomy are options for second-line treatment. In states of life-threatening acute and severe cortisol excess, the use of the cortisol-suppressive agent etomidate has been effective in controlling cortisol so that definitive treatment such as adrenalectomy can be performed. The above therapies with their indications, results and consequences, will be discussed individually below.

### Transsphenoidal pituitary surgery

Mainly developed in the paediatric setting by Charles Wilson at the University of California, San Francisco, in the 1980s [73, 74], TSS with selective microadenomectomy is the first-line treatment in paediatric CD, due to the high prevalence of corticotroph microadenomas [4, 68, 71]. If successful, this approach will result in complete tumour resection and disease remission [71]. However, this surgical technique is challenging, with relatively few pituitary surgeons having long-standing experience of operating in children. Some technical aspects warrant consideration, namely the anatomy of the sellar region varying with age, and the fact that the sphenoid bone, usually solid at birth, undergoes pneumatisation from age 2 years until adolescence, limiting the identification of bony landmarks necessary for safe surgery [75]. Also, the inter-carotid distance is usually shorter in children aged less than 7 years compared to adults. Finally, paediatric patients with skull base lesions, such as pituitary tumours, present specific anatomic variants, including shorter nare-sellar and vomer-clivus distances and smaller transsphenoidal angles [75].

Due to the specific features of paediatric pituitary surgery, TSS in CD paediatric patients should be performed in a small number of specialist centres with high surgical volumes and by neurosurgeons with TSS experience in children [68, 71]. Total excision of a corticotroph adenoma results in immediate post-operative ACTH and cortisol deficiency [68]. Normal corticotroph cells surrounding the adenoma are suppressed and undergo morphological alterations known histologically as Crooke’s change [68].

#### Remission following TSS

There is no international consensus on the definition of successful TSS outcome. It is generally agreed that the term ‘remission’ is more appropriate than ‘cure’ [71]. Remission is generally defined as morning serum cortisol values <5 μg/dL (<138 nmol/L) or urinary free cortisol (UFC) <28–56 nmol/day (<10–20 μg/day) within 7 days of selective tumour resection [71].

Two series of TSS in paediatric CD patients used a strict criterion for remission, namely post-TSS serum cortisol of <1 μg/dL [28 nmol/L] [40] or < 1.8 μg/dL [50 nmol/L] [4] and reported remission rates of 100 and 69%, respectively. Other series reported remission rates of 70–98% [36, 76]. An Indian series of 48 patients reported an overall cure rate of 56% with 77% cure for microadenomas [33]. When the patients treated at the NIH were analysed according to ethnic group, a higher proportion of subjects of Hispanic/Latino or African-American background, experiencing more severe CD features at presentation, had lower rates of initial cure and higher recurrence (10 out of 35 AA/HI, 28.6%) when compared to non-Hispanic white subjects (8 out of 78, 10.3%, *p* = 0.024) [77]. Successful TSS for paediatric CD is, however, associated with pituitary hormone deficiencies. Results from the NIH demonstrated GHD in a significant proportion of cases [78].

A German personal series of 100 children with CD operated on using the trans-nasal approach has recently been reported [68]. Over a 30-year period from 1980 to 2009, remission rates of 100% from 1980 to 1995 and 98% from 1996 to 2009 were achieved (63).. In a further recent Italian series of 43 patients with paediatric CD, a remission rate of 72.1% was reported [79]. The most common complication of TSS was post-operative diabetes insipidus, present in 5% of subjects at discharge from neurosurgical care [36].

In summary, TSS is effective and safe first-line treatment for paediatric CD, although disease persistence may occur in up to approximately 30% of treated patients, who require second-line therapy which can be re-operation (TSS), pituitary RT, medical therapy to control cortisol synthesis, or bilateral adrenalectomy.

#### Endoscopic TSS

Another modification of the TSS approach is the endoscopic technique, using either the trans-nasal or endonasal approach. This form of TSS appears to be less invasive than the classical sub-labial approach and is now the established technique of many pituitary surgeons for adult patients. In paediatric CD, a British series of 6 CD children aged 11 to 17 years reported that endoscopic endo-nasal TSS resulted in biochemical remission in 5, with no recurrence reported after a mean follow-up of 55 months [80]. Transient diabetes insipidus occurred in 5/6 subjects. In a recent multicentre paediatric study from Italy and Turkey, 4/5 patients with CD achieved remission, one needing repeat endoscopic TSS [81].

#### Recovery of the hypothalamic-pituitary-adrenal (HPA) axis

The adrenal insufficiency (AI) which follows complete removal of an ACTH-secreting adenoma requires physiological glucocorticoid replacement with hydrocortisone 8–12 mg/m^2^/day, and may persist for many months. A recent study from the NIH looked at factors potentially influencing the duration of AI and recovery of the HPA axis [82]. In 102 paediatric CD patients who recovered adrenal function following post-TSS remission, the median time to recovery was 12.3 months with a range of 3–35 months. The only biochemical variable at diagnosis related to recovery time was UFC, in which higher pre-operative values were associated with a longer interval to recovery of normal adrenal function, assessed by standard ACTH stimulation testing. Previous reports on factors predicting recovery of the HPA axis have been inconsistent [72]. The NIH study also reported, for the first time in paediatric CD, a statistically significant relationship (*p* = 0.0342) between a shorter HPA axis recovery time, and the likelihood of recurrence of CD [82], as had been shown in adults [83]. All patients with recurrence of hypercortisolism had recovery of the axis by 15 months post-TSS.

### Pituitary radiotherapy (RT)

As discussed above, a minority of children receiving TSS for paediatric CD will not achieve remission and second-line therapy is needed to prevent continuing hypercortisolism. External pituitary RT is a therapeutic option in such patients [71, 84]. Unlike evidence in adult CD patients, few reports are available in children. Considering that over 90% of paediatric CD patients have microadenomas [4, 68], conventional fractionated external RT has been used rather than other techniques. However, pituitary RT is not universally practised in children, largely related to concern about effects on cognitive function [36].

In paediatric CD patients, gamma knife stereotactic radiosurgery was reported in 24 patients aged 10 to 21 years from a multi-national study [85]. Although precise details of the adenomas are not given, 18/24 patients had previously been treated unsuccessfully with TSS and 8 had cavernous sinus invasion by the adenoma, suggesting that these were not typical corticotroph microadenomas. Remission of the hypercortisolism occurred in 87.5% of subjects after a mean interval of 12 months with 20% developing new pituitary hormone deficiencies related to the RT. [85]

The rapid response of corticotroph adenomas to external RT was first documented in 1977 [86]. In 1985, external RT was delivered to 8 paediatric patients using a stereotactic technique delivering 50–70 Gy and inducing remission in 88% [87]. These findings were confirmed in a report from London in 7 patients, treated by a 6-MV linear accelerator delivering a dose of 45 Gy in 25 fractions over 35 days [88]. Remission from hypercortisolaemia occurred at a mean interval of 0.94 years (range 0.25–2.86) [88]. In a subsequent Indian paediatric series of 8 patients, external RT induced a 50% remission rate, with 2 further patients having dexamethasone-suppressible cortisol at 26 months post-irradiation [89]. RT therefore takes several months to be effective and medical therapy such as ketoconazole will be required to control hypercortisolism until circulating cortisol decreases to within the physiological range [72, 88].

#### Pituitary function following radiotherapy

Anterior pituitary hormone deficiencies may occur following pituitary RT for CD [71, 72, 84]. Therefore, long-term pituitary function should be monitored and investigated. However, the degree of individual hormone deficiencies is variable. Short-term GHD occurred most commonly [85–87, 89], being present in 86% of patients reported by Storr et al. Follow-up for a mean period of 10 years in the same patients showed that 3/4 boys regained normal GH secretion (Peak GH >10 ng/ml) [46]. It may be incorrect to attribute anterior pituitary deficiencies to RT alone because TSS induces GHD [78]. The long-term follow-up of 20 patients treated by TSS (*n* = 15) or TSS + RT(*n* = 5) for a mean interval of 10.6 years showed some recovery of GH secretion, but with 44% having long-term adult GHD [47]. Gonadotrophin deficiency was also present in 9/20 subjects causing impaired pubertal development and 4 requiring sex steroid replacement post-puberty [47].

### Medical treatment

Medical treatment aimed at reducing circulating cortisol is usually a second-line therapy or potential first-line treatment in patients unwilling or unable to undergo TSS or pituitary RT [71, 72]. Medical therapy aims to suppress adrenal steroidogenesis and can be divided into potential long-term therapy such as ketoconazole, mitotane and metyrapone and shorter-term therapy such as etomidate. There are no substantive paediatric studies on cabergoline, mifepristone, osilodrostat or pasireotide.

Ketoconazole impairs adrenal and gonadal steroidogenesis by inhibiting side-chain cleavage, 17,20-lyase, and 11-β hydroxylase enzymes. There are anecdotal reports in children, but but no definitive trial has been published. The main indications for ketoconazole are when awaiting TSS, after unsuccessful TSS, and during the period after RT and before normalisation of cortisol. A recent French report described treatment of 9 paediatric CD patients with ‘low-dose’ mitotane (titrated from 1 g/day to normalise UFC levels) [90]. After 12 months of therapy, height velocity and BMI SD scores improved significantly with changes comparable to those seen in 13 matched subjects in remission following TSS. Mitotane improved some features of CD but without perfect control of hypercortisolism. The adverse effects included digestive symptoms, weakness and AI, which were dose-dependent, making this therapy largely inappropriate for long-term therapy in children [90]. Monitoring of cortisol levels is also problematic.

There are acute clinical situations, such as the association of severe hypercortisolaemia with complications such as respiratory failure or acute psychosis, where rapid control of cortisol secretion is necessary in order to allow emergency therapy such as bilateral adrenalectomy. Intravenous etomidate infusions administered in an intensive care environmentat doses ranging from 1 to 3.5 mg/h have been documented to be effective in suppressing hypercortisolaemia in three paediatric CD patients [44, 46, 91].

### Bilateral adrenalectomy

Bilateral adrenalectomy is an important therapeutic option which may be life-saving and should be considered when first-line therapy is not available or when the clinical state of the patient does not allow TSS or pituitary RT, or when medical therapy fails to control hypercortisolaemia [71]. Adrenalectomy will eliminate hypercortisolaemia and reverse related complications rapidly [92]. However, it requires life-long replacement with glucocorticoid and mineralocorticoid therapy and does not remove the primary cause of CD, which remains in situ as a corticotroph adenoma [5, 72].

### Nelson’s syndrome

Nelson’s syndrome may occur after bilateral adrenalectomy and is defined as MRI evidence of macroscopic (>1 cm) corticotroph tumour enlargement associated with increasing levels of ACTH causing hyperpigmentation [93]. Consequently, after bilateral adrenalectomy, paediatric patients require regular evaluation using pituitary MRIs and ACTH levels.

There are anecdotal reports of Nelson’s syndrome following bilateral adrenalectomy in children with CD [47] but few published series. In 6 children who underwent adrenalectomy between 8 and 17 years of age, 4 developed Nelson’s syndrome at 2, 6, 10, and 12 years post-adrenalectomy. A review of the literature showed that in 37 patients with Nelson’s syndrome, the mean age at diagnosis of CD was 12 years, with a mean interval of 8.4 years post-adrenalectomy [94]. In an earlier study, 31 patients aged from 10 months to 16 years underwent bilateral adrenalectomy for CD and post-adrenalectomy hyperpigmentation was reported in 18 patients with sella enlargement in 8 patients (25%) 1 to 5.5 years post-adrenalectomy [95]. Consequently, Nelson’s syndrome is a genuine risk in the paediatric age range and life-long follow-up is necessary. Nelson’s syndrome, if it occurs, should be managed according to current adult endocrine practice [71, 96].

## Recurrence of Cushing’s disease after remission

There have been few studies which have formally looked at paediatric recurrence. Follow-up data after TSS and remission suggested that recurrence of hypercortisolism was uncommon [4, 32]. Yordanova et al. studied long-term follow-up of 21 paediatric CD cases in remission following definitive therapy. The recurrence rate was 14.3% occurring from 2 to 7.6 years after primary treatment [47]. The large series of patients treated at the NIH (*n* = 72) has been studied for short-term and long-term remission and recurrence of CD [32, 36]. Children who remained in remission following TSS had significantly lower morning ACTH and cortisol levels compared with those who relapsed. Also, during a CRH stimulation test, ACTH and cortisol values were higher in patients who relapsed compared with those in remission. Relapse was associated with lack of histological confirmation of an adenoma, normal serum cortisol or ACTH and a normal response to CRH [32]. An expanded data-set of the NIH patients (*n* = 200) was reported by Lonser et al. in 2013, of whom 179 were available for longer term analysis. At 5 years post-TSS, 96.7% and at 10 years 90.5% were free of recurrence [36]. The study also associated long-term remission with younger age, smaller adenoma, and morning serum cortisol of <1 μg/dL after surgery [36]. The current recurrence rate of paediatric patients treated at the NIH is 11.5% at a median of 44 months (range: 6.3–97 months) after TSS [82].

## Complications after induction of remission

### Linear growth

In patients experiencing growth failure due to endogenous hypercortisolism, achievement of target height during post-surgical remission depends on several hormonal and clinical factors. Elements limiting catch-up growth are onset of CD during puberty [34], advanced bone age [97] and the presence of vertebral fractures.

GHD following TSS has been clearly demonstrated [78] and is also seen following external fractionated [89, 98] or stereotactic [87] pituitary RT. Adult height in CD patients in remission is significantly below target height [99, 100]. We advocate testing for GHD 3 months into biochemical remission with a low threshold for GH replacement therapy [55]. Although no controlled data are available, the combined use of GH and GnRH analogues in patients with GHD and severe short stature during puberty in order to delay bone age progression supports this therapeutic approach [55, 101].

### Long-term pituitary function

GHD lasting for several years and persisting into adult life has been reported after successful therapy of CD using TSS or pituitary RT. [98] The degree of GHD was variable, being more severe in subjects with additional anterior pituitary deficiencies [98]. The long-term effects of successful pituitary RT were reported in 6 male subjects. At a mean of 1.0 year (0.11–2.54) after RT, 5 subjects (84%) had GHD with peak GH <10 ng/ml, but when reassessed at a mean of 9.3 years (7.6–11.3) after RT, 75% had normal GH secretion, i.e. Peak GH >9.6 ng/ml. Other anterior pituitary functions in 5/6 patients were normal on follow-up. All the six patients had testicular volumes of 20–25 ml at the age of 14.5–28.5 years [102]. A longer-term follow-up was reported in 21 patients, some also included in the previous report, after a mean interval from definitive treatment of 10.6 years (2.9–27.2) [47]. Gonadotropin deficiency caused impaired pubertal development in 9 patients (43%), 4 requiring sex steroid replacement post-puberty and 4 patients (19%) had more than one pituitary hormone deficiency, 3 after TSS and one after RT. Pituitary deficiencies at diagnosis and during remission of CD are described in Table 4**.**

**Table 4:** Auxology, body composition and pituitary deficiencies at diagnosis and during remission of Cushing’s disease following therapy with transphenoidal surgery or pituitary radiotherapy

Centre	Treatment	F/M	Pubertal status at diagnosis	Height SDS		BMI SDS		Bone mineral density Z-Score		Pituitary hormone deficiencies
				diagnosis	latest assessment	diagnosis	latest assessment	diagnosis	latest assessment	
Barts Health, London, UK [47]	1st line: TSS (n = 21); 2nd line: RT (n = 5)	(8/13)	pre-pubertal 8 (4 M; 4F); post-pubertal 13 (9 M;4F) (n = 21)	−1.5 (0.1 to −3.3) (n = 21)	−0.99 (0.5 to −2.9) (n = 21; *6.7 y*)	2.9 (0.1 to 6.9) (n = 21)	0.58 (−1.9 to 2.7) (n = 21; *6.7 y*)	LS -1.3 (−0.3 to −2.6) (*n* = 5); L2-L4–2.0 (−0.6 to −3.3) (n = 5); FN -1.7 (−1.0 to −2.1) (*n* = 3)	L2-L4–0.5 (1.1 to −2.0) (n **= 9**; 5.2 y) FN 0.22 (2.46–0.9) (n = 5; *5.8 y*)	4/21 (16.7%) AI, 2/21 (8.3%) DI, 14/19 (81%) GH, 4/21 (16.7%) GT, 4/21 (16.7%) TSH (n = 21; *6.7 y*)
NIH, Bethesda, USA [77, 97, 99, 103]	1st line: TSS (*n* = 129); 2nd line: RT (n = 2); ketoconazole (n = 3) [77]	(65/64) [77]	pre-pubertal 9 (8 M;1F); post-pubertal 41 (30F;11M) (n = 50) [99]	−1.19 ± 1.1 (n = 35) [97]	−0.81 ± 1.2 (n = 16; *1.2 y*) [97]	1.65 ± 0.4 (*n* = 7) [97]	0.88 ± 0.3 (n = 7; *7 y*) [97]	LS -1.6 ± 1.37 FN -1.04 ± 1.19 (n = 35) [97]	LS -0.66 ± 1.55 FN -0.79 ± 1.35) (n = 16, *1.2 y*) [97]	3/37(8%) DI; 3/37 (8%) GH; 1/37 (3%) GT; 6/37 (16%) TSH (*n* = 37; *1.8 y*) [99]
UCSF, USA [34]	1st line: TSS (*n* = 42); 2nd line: repeat TSS (*n* = 8); BADX (n = 4)	(25/17)		−1.8 (+0.3 to −3.5) (n = 42)	−1.14 (−2.5 to −0.7) (n = 20; *7.2 y*)					2/20 (10%) AI; 4/20 (20%) GH; 1/20 (5%) TSH; (n = 20; *7.2 y*)
Mumbai, India [89, 104]	1st line: TSS (*n* = 48); 2nd line: RT (*n* = 11); repeat TSS (n = 7); BADX (n = 4) [89]	(19/29) [104]	pre-pubertal 2; post-pubertal 31; pseudo-puberty 15 (n = 48) [104]	−1.9 (−0.3 to −6.7) (n = 48) [104]	−1.84 (+0.6 to −6.7) (n = 20; *4.1 y*) [104]					1/8 (12.5%) GH; 2/8 (40%) GT; 1/8 (12.5%) TSH (n = 8; *5 y*) [89]
Naples, Italy [105]	1st line: TSS (*n* = 6);	(3/3)	pre-pubertal 0; post-pubertal 6 (3F;3M) (n = 6)					LS -2.59 ± 0.4 (n = 6)	LS -2.22 ± 0.3 (n = 6; *2y*)	

Data are presented as: mean ± SD or (range) (number of patients; mean follow up in years); *TSS* Transphenoidal surgery, *RT* Pituitary radiotherapy, *BADX* Bilateral adrenalectomy, *M* Male, *F* Female, *y* Year/s, *BMI* Body mass index, *LS* Lumbar spine, *FN* Femur neck, *AI* Adrenal insufficiency, *DI* Diabetes insipidus, *GH* Growth hormone, *GT* Gonadotropin, *TSH* Thyroid stimulating hormone

### Body composition: Body mass index and bone mineral density

Paediatric patients with CD show increased body weight and BMI at diagnosis [4]. In a study of 59 patients with CS due to multiple aetiologies from the NIH, BMI decreased to normal by 1 year following successful definitive therapy [106]. However, in patients with CS presenting around the age of adolescence, BMI remained elevated, notably in female patients [103]. Elevated BMI after remission was reported in a 15-year-old patient who was followed for 6 years after remission and body composition was compared to that of her normal co-twin [107] with total body fat, abdominal visceral fat, and subcutaneous fat (%) being 42, 10, and 41 in the CD subject versus 26, 4, and 17 in the normal twin. In 14 CD patients followed to adult height after biochemical remission BMI SDS was +1·7 (0·4–6·2), being decreased compared to diagnosis (*P* < 0·05) but remaining greater than the normal population (P < 0·01) [55].

Osteoporosis is a common complication in paediatric CD, affecting trabecular bone more severely than cortical bone [108]. Results have shown a significant increase in bone mineral density (BMD) during remission. In 14 subjects with CD followed for a minimum of 3 years post-remission, BMD and apparent density (BMAD) SDS at the lumbar spine (LS) at diagnosis were − 1.8 and − 1.25, respectively, and after 3 years of follow-up approached the mean with no further increase apparent up to 7 years of follow-up [103]. Whereas hip BMD SDS increased from −1.3 at diagnosis to −0.40 at 3 years and 0 at 7 years of follow-up, femoral neck BMAD remained at or around 0 SDS at diagnosis and during follow-up [103].

In children with CD, vertebral BMD was more severely affected than femoral BMD being independent of the degree or duration of hypercortisolism. Lumbar spine BMD improved significantly after TSS [109]. Deficits in BMD and serum osteocalcin levels in childhood-onset CD patients were partly, but not completely, reversed 2 years after normalisation of cortisol levels, although longer recovery times or additive therapy, such as bisphosphonates, may be necessary to maximize peak bone mass in children and adolescents [105]. Auxology data before and after treatment are shown in Table 4**.**

### Blood pressure, metabolic variables and psychological status

Detailed studies on blood pressure (BP) have been performed at the NIH [106]. Approximately half of children and adolescents with CD develop hypertension [4]. The role of hypercortisolism in the pathogenesis of hypertension and its reversibility was studied in 31 hypertensive children and adolescents with CS of various aetiologies before and for a period of 1 year after surgical cure [106]. Preoperatively, 45% of these patients presented with an increase of mean BP. Following normalisation of cortisol, systolic BP remained increased in 30.7%, 15.8%, and 5.5% of patients at 3, 6, and 12 months respectively whereas diastolic and mean BP normalised by 3 months after surgical cure. A significant, positive correlation was observed between the systolic BP and the duration of the disease, pointing towards the deleterious effects of prolonged hypercortisolism. The normalisation of BP within a year from the correction of hypercortisolism suggests that, in general, young patients with CD do not develop essential hypertension [106]. However, in approximately 4% of patients, residual hypertension persisted after normalisation of cortisol [110].

Studies in metabolic parameters have shown a significant improvement in biochemical variables, such as fasting glucose, fasting insulin, HOMA index and lipid profile during remission [111]. As described above, central obesity is likely to persist with a positive correlation reported between waist circumference and fasting insulin 1 year after remission [111].

Psychological abnormalities and decreased quality of life contribute to the complex symptomatology of paediatric CD. The association between hypercortisolism and abnormalities in hippocampal volume and cognitive function has been described, although it is not clear to what extent this applies to children with CD [112]. However, cerebral atrophy has been documented in paediatric CD and although this improves rapidly during remission, decreased cognitive function and school performance were still evident 1 year after successful remission [113]. Specific psychological disturbances have recently been described in children after surgical cure of CS. These include behavioural changes such as anger-rage outbursts and affective symptoms such as suicidal ideation, anxiety and depression [114]. Clearly, the central nervous and psychological effects of prolonged hypercortisolism are potentially profound and awareness of their possible occurrence forms part of the long-term management of CD in remission.

## Conclusions

The rarity of paediatric CD presents challenges in referral and diagnosis because the pathological nature of the clinical features may not be recognised until they are extreme. By that time morbidity affecting growth, puberty, body composition, bone mineral density and psychological status may be established and difficult to reverse. The importance of early diagnosis reflects the urgency of controlling hypercortisolism to prevent further complications. As mentioned above, close consultation between paediatric and adult endocrinologists is strongly recommended to optimise clinical care. Therapeutic options are essentially the same as for adult CD, however the choice of a pituitary surgeon with experience of TSS in children is essential. Even in the best hands, post-surgical remission in all patients is unlikely and opinions on second-line therapy are likely to vary between countries and institutions. Paediatric CD patients, managed in experienced units, have a good prognosis, although on-going challenges in terms of normalisation of disturbed growth, pituitary function, BMI, BMD and psychological problems may remain. The possibility of recurrence of hypercortisolism will necessitate long-term follow-up with eventual transition to a specialist adult endocrine department.

## Data Availability

Not applicable.
